# Supplementary figures and images for: Immuno-oncologic profiling by stage-dependent transcriptome and proteome analyses of spontaneously regressing canine cutaneous histiocytoma
Source: PeerJ. 2024 Nov 26;12:e18444. doi: 10.7717/peerj.18444 (PMC11606323; doi:10.7717/peerj.18444)

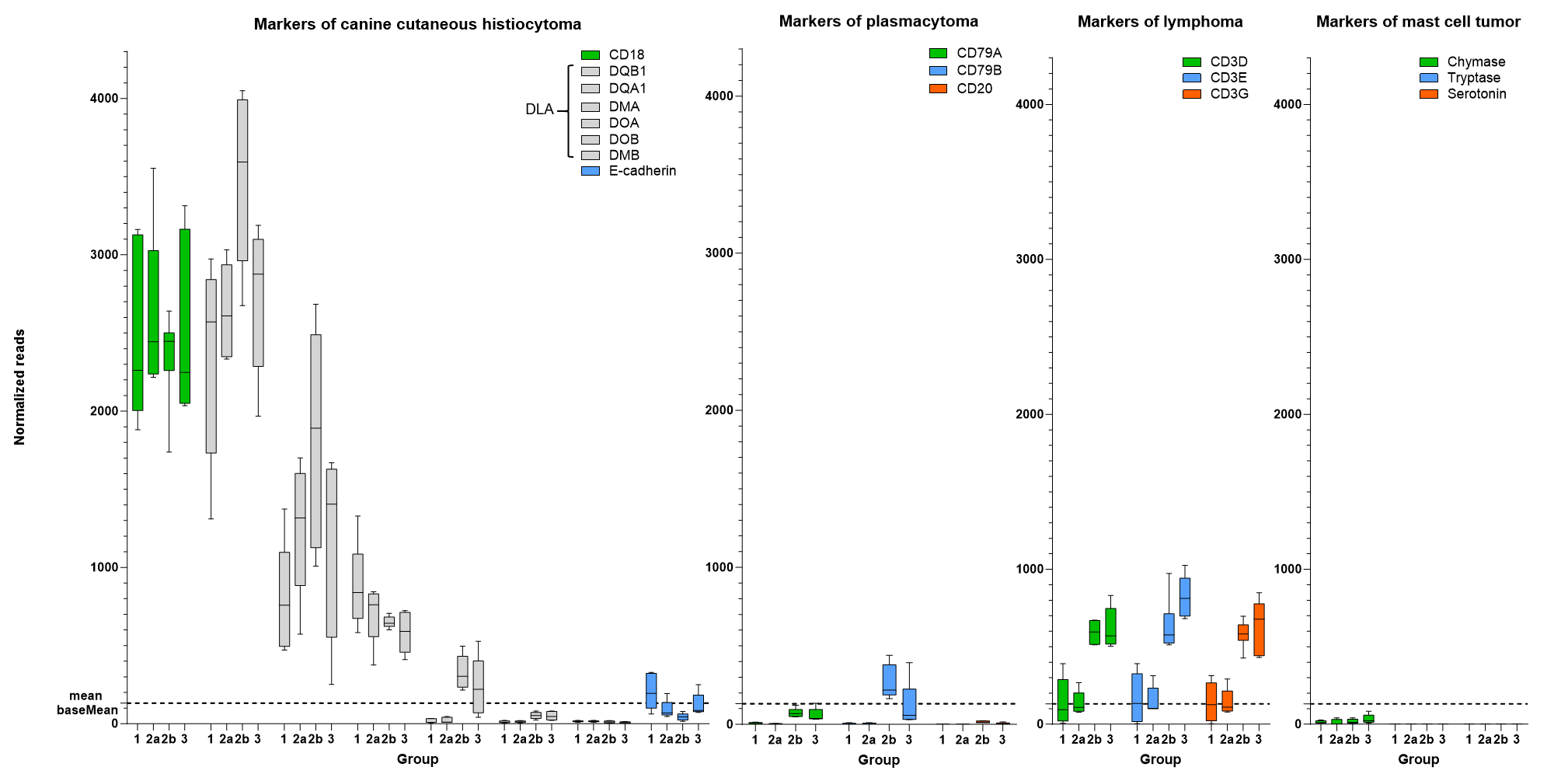

Supplement: Supplemental Information 1 — Box-and-whisker plots display values as maximum, minimum, median, lower and upper quartiles (n = 5 or 6) of normalized counts as detected by QunatSeq 3′ analysis. The mean baseMean of all detected RNAs (132, dashed line) is provided as a basis for comparison to the number of respective round cell tumor marker counts. [file peerj-12-18444-s001.png]

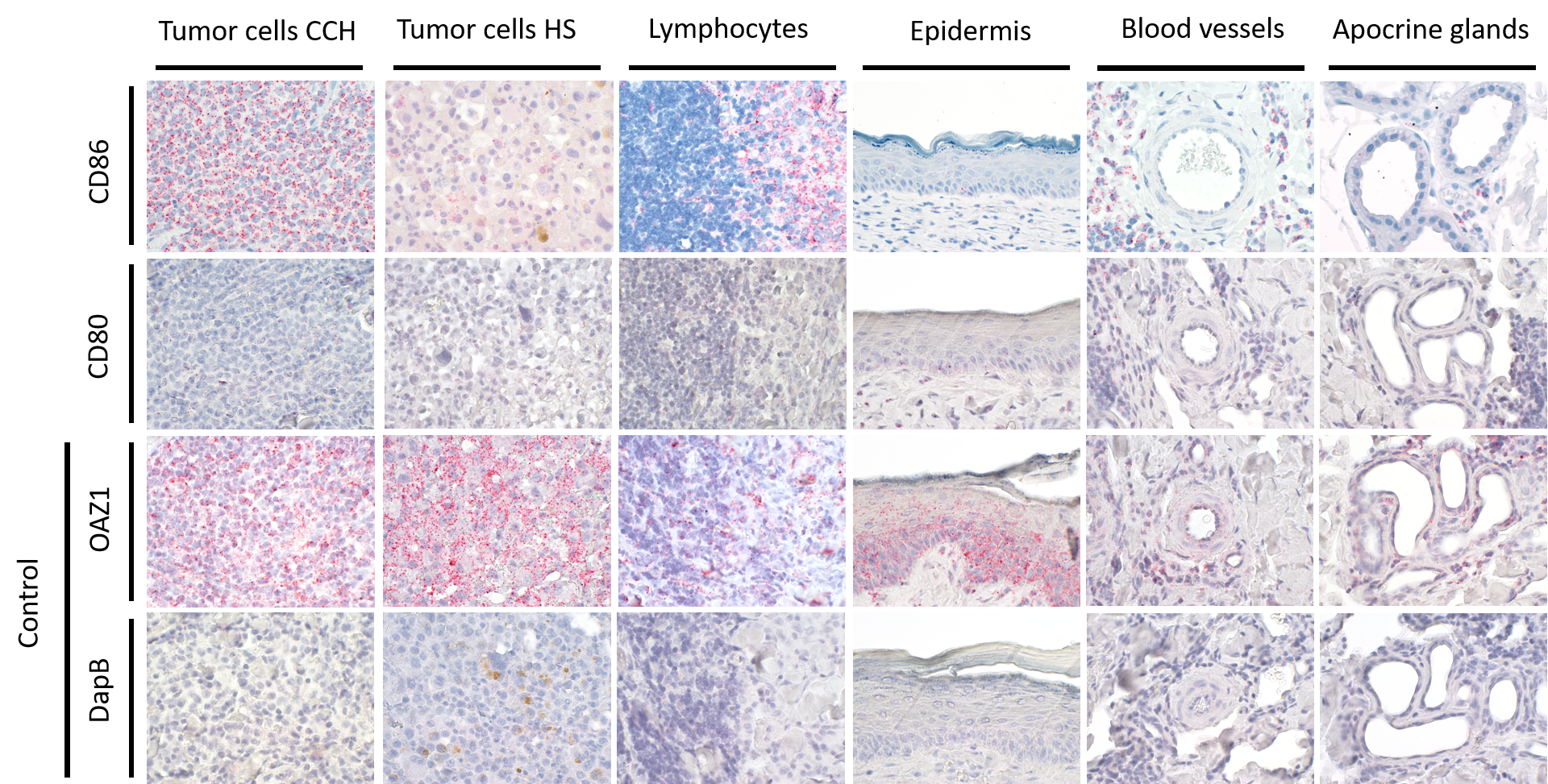

Supplement: Supplemental Information 2 — Ornithine decarboxylase antizyme 1 (OAZ1) and dihydrodipicolinate reductase (DapB) in tumor cells of canine cutaneous histiocytoma (CCH) and canine histiocytic sarcoma (HS), as well as lymphocytes, epidermis, blood vessels and apocrine glands of tumor adjacent tissue of CCH. OAZ1 served as control for RNA accessibility. DapB served as a negative control. I. situ hybridization with fast red (chromogen, red) and Mayer’s hematoxylin (blue) counterstain. Magnification: 600×. [file peerj-12-18444-s002.png]
