# Supplementary material for: Immuno-oncologic profiling by stage-dependent transcriptome and proteome analyses of spontaneously regressing canine cutaneous histiocytoma
Source: PeerJ. 2024 Nov 26;12:e18444. doi: 10.7717/peerj.18444 (PMC11606323; doi:10.7717/peerj.18444)
Supplement: Supplemental Information 3 [file peerj-12-18444-s003.docx]

| **Samples used for transcriptome analysis** | | | | | | | |
| --- | --- | --- | --- | --- | --- | --- | --- |
| **Sample ID_group** | **RQN** | **DV200**  **(%)** | **breed** | **age (years)** | **sex** | **location** | **size**  **(up to X cm)** |
| AL3_1 | 6.3 | 68.9 | French Bulldog | 0.5 | mn | Skin paw | 1.5 |
| AL4_1 | 4.1 | 68.3 | Goldendoodle | 0.75 | m | Skin shoulder | 1.1 |
| AL6_1 | 7.7 | 71.2 | Terrier | n/s | n/s | Skin neck | 1.0 |
| AL7_1 | 7.8 | 69.4 | Boxer | 5.0 | mn | Skin ear | 1.1 |
| AL8_1 | 4.5 | 64.5 | French Bulldog | 3.0 | f | Skin thigh | 1.7 |
| AL17_2a | 7.4 | 76.6 | Bavarian Mountain Hound | 0.6 | m | Skin lip | 2.1 |
| AL22_2a | 8.3 | 70.5 | English Bulldog | 3.0 | mn | Skin paw | 1.5 |
| AL24_2a | 8.8 | 76.2 | Hovawart | 3.0 | w | Skin leg | 1.9 |
| AL25_2a | 7.6 | 71.7 | Doberman | 1.0 | wn | Skin ear | 1.1 |
| AL28_2a | 8.9 | 73.7 | Mix | 1.5 | m | Skin carpus | 1.7 |
| AL33_2b | 6.9 | 76.3 | Akita | 0.7 | w | Skin leg | 1.5 |
| AL34_2b | 5.4 | 72.5 | Pug | 6.0 | wn | Skin face | 1.2 |
| AL35_2b  (pooled sample) | 5.2 | 76.9 | Rhodesian Ridgeback | 1.0 | w | Skin flank | 1.2 |
|  |  |  | French Bulldog | 3.0 | w | Skin chest | 1.1 |
|  |  |  | French Bulldog | 0.8 | m | Skin abdomen | 1.3 |
|  |  |  | Dachshund | 2.0 | m | Skin loin | 1.1 |
| AL39_2b (pooled sample) | 4.7 | 73.1 | Doberman | 0.8 | m | Skin flank | 2.1 |
|  |  |  | French Bulldog | 3.0 | mn | Skin abdomen | 1.2 |
|  |  |  | Jack Russel Mix | 1.0 | w | Skin face | 1.4 |
|  |  |  | n/s | n/s | n/s | Skin lip | 1.4 |
| AL40_2b  (pooled sample) | 5 | 74.3 | Flat Coated Retriever | 1.7 | w | Skin leg | 1.5 |
|  |  |  | n/s | 0.5 | m | Skin shoulder | 1.1 |
|  |  |  | Boxer | 3.5 | mn | Skin n/s | 1.4 |
|  |  |  | Bernese Mountain Dog | 1.5 | w | Skin paw | 2.4 |
| AL45_2b (pooled sample) | 5.7 | 74.7 | Bordeaux Mastiff | 3.0 | m | Skin paw | 1.3 |
|  |  |  | French Bulldog | 4.0 | w | Skin face | 1.4 |
|  |  |  | Labradoodle | 0.5 | m | Skin leg | 2.0 |
| AL48_3 | 5.8 | 72.8 | Rottweiler | 1.5 | mn | Skin chin | 2.4 |
| AL51_3 | 7.2 | 74.8 | Beagle | 9.0 | f | Skin tarsus | 1.7 |
| AL52_3 | 8.3 | 75.8 | Pug | 2.5 | m | Skin flank | 1.2 |
| AL54_3 | 7.4 | 71.2 | Bullterrier | 2.0 | m | Skin thigh | 1.5 |
| AL57_3 | 6.7 | 74.5 | Jack Russell Terrier | 7.0 | m | Skin paw | 1.2 |

| **Samples used for *in situ* hybridization** | | | | | | | |
| --- | --- | --- | --- | --- | --- | --- | --- |
| **Sample ID** | **Group** | **Target** | **breed** | **age (years)** | **sex** | **location** | **size**  **(up to X cm)** |
| In situ_1 | CCH1 | CD86 | English Miniature Bullterrier | 0.75 | m | Skin n/s | 0.3 |
| In situ_2 | CCH1 | CD86 | French Bulldog | 1.0 | w | Skin n/s | 1.1 |
| In situ_3 | CCH1 | CD86 | Labrador-Golden Retriever | 6.0 | w | Skin paw | 2.2 |
| In situ_4 | CCH2 | CD86 | Goldendoodle | 3.75 | m | Skin thorax | 0.9 |
| In situ_5 | CCH2 | CD86 | Staffordshire | 7.0 | wn | Skin thorax | 1.8 |
| In situ_6 | CCH2 | CD86 | Cane Corso | n/s | mn | Skin ear | 1.1 |
| In situ_7 | CCH3 | CD86 | Great Dane | 2.0 | w | Skin lip | 2.5 |
| In situ_8 | CCH3 | CD86 | Mix | 10 | wn | Skin lip | 1.2 |
| In situ_9 | CCH3 | CD86 | Labrador Retriever | 2.0 | m | Skin face | 0.5 |
| In situ_10 | CCH1 | CD80 | Cane Corso | 0.5 | m | Skin thorax | 1.6 |
| In situ_11 | CCH1 | CD80 | French Bulldog | 1.0 | w | Skin n/s | 1.3 |
| In situ_12 | CCH1 | CD80 | Border Colli | 0.7 | m | Skin paw | 1.2 |
| In situ_13 | CCH2 | CD80 | French Bulldog | n/s | m | Skin leg | 1.4 |
| In situ_14 | CCH2 | CD80 | Labrador Retriever | 1.0 | m | Skin leg | 2.3 |
| In situ_15 | CCH2 | CD80 | Bordeaux Mastiff | 8.0 | mn | Skin lip | 1.4 |
| In situ_16 | CCH3 | CD80 | French Bulldog | 2.0 | w | Skin n/s | 3.7 |
| In situ_17 | CCH3 | CD80 | Mix | 4.0 | mn | Skin tarsus | 1.8 |
| In situ_18 | CCH3 | CD80 | Mix | 2.0 | mn | Skin loin | 1.7 |
| In situ_19 | HS | CD80, CD86 | Bernese Mountain dog | 9.0 | mn | Spleen | 7.7 |
| In situ_20 | HS | CD80, CD86 | Mix | 9.0 | mn | Spleen | 14.4 |
| In situ_21 | HS | CD80, CD86 | Small Münsterländer | 8.0 | wn | Knee n/s | 6.5 |

| **Samples used for MALDI-MSI** | | | | | | |
| --- | --- | --- | --- | --- | --- | --- |
| **Sample ID** | **Group** | **breed** | **age (years)** | **sex** | **location** | **size**  **(up to X cm)** |
| MALDI_1 | CCH1 | English Miniature Bullterrier | 0.75 | m | Skin n/s | 0.3 |
| MALDI_2 | CCH1 | Labrador-Golden Retriever | 6.0 | w | Skin paw | 2.2 |
| MALDI_3 | CCH1 | French Bulldog | 1.0 | w | Skin n/s | 1.1 |
| MALDI_4 | CCH2 | Staffordshire | 7.0 | wn | Skin thorax | 1.8 |
| MALDI_5 | CCH2 | Goldendoodle | 3.75 | m | Skin thorax | 0.9 |
| MALDI_6 | CCH2 | Pug | 1.0 | m | Skin n/s | 1.0 |
| MALDI_7 | CCH3 | Mix | 10 | wn | Skin lip | 1.2 |
| MALDI_8 | CCH3 | n/s | n/s | n/s | n/s | 1.5 |
| MALDI_9 | CCH3 | Great Dane | 2.0 | w | Skin lip | 2.5 |

m: male

f: female

mn: male neutered

fm: female neutered

n/s: not specified
