# Supplementary material for: Immuno-oncologic profiling by stage-dependent transcriptome and proteome analyses of spontaneously regressing canine cutaneous histiocytoma
Source: PeerJ. 2024 Nov 26;12:e18444. doi: 10.7717/peerj.18444 (PMC11606323; doi:10.7717/peerj.18444)
Supplement: Supplemental Information 5 [file peerj-12-18444-s005.docx]

| Gene Sets |
| --- |
| Adhesion |
| Angiogenesis |
| Antigen Presentation |
| Antigen Processing |
| Apoptosis |
| Autophagy |
| B Cell Functions |
| Cell Cycle |
| Cell Functions |
| Cell Proliferation |
| Chemokines |
| Complement |
| Complement System |
| Costimulatory Signaling |
| Cytokine and Chemokine Signaling |
| Cytokines |
| Cytotoxicity |
| DNA Damage Repair |
| Epigenetic Regulation |
| Hedgehog Signaling |
| Hypoxia |
| Immune Cell Adhesion and Migration |
| Interferon Signaling |
| Interleukins |
| JAK-STAT Signaling |
| Leukocyte Functions |
| Lymphoid Compartment |
| Macrophage Functions |
| MAPK |
| Matrix Remodeling and Metastasis |
| Metabolic Stress |
| Microglial Functions |
| Myeloid Compartment |
| NFkB Signaling |
| NK Cell Functions |
| Notch Signaling |
| Pathogen Defense |
| PI3K/Akt |
| Regulation |
| Senescence |
| T Cell Functions |
| TGF-beta Signaling |
| TLR |
| TNF Superfamily |
| Wnt Signaling |
